# Changes in Waveguiding Cone Photoreceptors and Color Vision in Patients With Diabetes Mellitus

**DOI:** 10.1167/iovs.65.14.28

**Published:** 2024-12-13

**Authors:** Megan Vaughan, Nicole Tay, Angelos Kalitzeos, Thomas Kane, Nav Singh, Adrian Zheng, Mira Dixit, Bishwanath Pal, Ranjan Rajendram, Konstantinos Balaskas, Mari Pilar Martin Gutierrez, Jose Carlo Artiaga, Georgios Koutsocheras, Khadra Adan, Marisa Rodriguez-Carmona, John L. Barbur, Michel Michaelides, Emily J. Patterson

**Affiliations:** 1 UCL Institute of Ophthalmology, University College London, London, England, United Kingdom; 2 Moorfields Eye Hospital NHS Foundation Trust, London, England, United Kingdom; 3 UCL Medical School, University College London, London, England, United Kingdom; 4 Department of Ophthalmology and Visual Sciences, Philippine General Hospital, University of the Philippines Manila, Manila, Philippines; 5 City, University of London, London, England, United Kingdom; 6 Occuity, Reading, United Kingdom

**Keywords:** diabetic retinopathy (DR), adaptive optics, retinal imaging, color vision, diabetes mellitus (DM)

## Abstract

**Purpose:**

Although it is well known that photoreceptor damage and color vision loss occur in patients with diabetic retinopathy (DR), the relationship between structural and functional changes in diabetes mellitus (DM) remains unclear. Using highly sensitive measures of photoreceptor structure and function, we aim to determine whether early loss of color sensitivity in DM is also accompanied by decreased cone density.

**Methods:**

Monocular data from 26 patients with DM and 25 healthy controls were examined to assess cone photoreceptor metrics, using confocal adaptive optics scanning light ophthalmoscopy, and red/green (RG) and yellow/blue (YB) color vision thresholds, using the Colour Assessment and Diagnosis test.

**Results:**

Both RG and YB thresholds were significantly greater in patients with DM than in the healthy controls (RG and YB = *P* < 0.001), and there were statistically significant differences between the 2 groups in confocal cone density at 1 degree (*P* = 0.024), and intercell regularity at both 1 (*P* = 0.013) and 2 degrees (*P* = 0.012). In patients with DM, cone density was inversely correlated with YB (at 0.5, 1 and 2 degrees, all *P* values < 0.041), but not for RG color vision thresholds.

**Conclusions:**

This is the first study to investigate the relationship between cone metrics and color vision in patients with DM. The results reveal a significant inverse relationship between confocal cone density and color vision thresholds at the locations assessed within the foveal region. These findings represent a significant advancement in oculomics research.

Diabetes mellitus (DM) affects approximately 463 million people worldwide,^1^ and it has been postulated that this will increase to 700 million people by the year 2045.^2^ One of the many negative associated health outcomes of DM is diabetic retinopathy (DR), which is a leading cause of vision loss in working-age adults in the developed world.^3^ Patients are diagnosed as having DR once they have at least one of the following: microaneurysms, hemorrhages, cotton wool spots, exudates, edema, or venous abnormalities.^4^ By the time these features become visible using fundus photography, it is often the case that damage has already afflicted the retina, which can be irreversible.^5^ Therefore, early diagnosis is key to preserving vision and preventing vision loss.

Adaptive optics scanning light ophthalmoscopy (AOSLO) enables visualization of individual photoreceptors in the human retina in vivo.^6^ AOSLO also offers the ability to focus selectively at different depths, enabling visualization of different retinal layers. Confocal AOSLO detects light transmitted through a pinhole, whereas split-detection AOSLO detects light that is multiply scattered by the retina. The simultaneous acquisition of both modalities allows direct comparison between reflective/dark areas within the confocal image and underlying structure in the non-confocal image. Using AOSLO, Tam et al.^7^^,^^8^ demonstrated changes in retinal microvasculature in DR. Karst et al.^9^ used both confocal and split-detection AOSLO to evaluate the appearance of the inner retinal layers within lesions, and found changes in the thickness of vessel walls, as well as abnormal reflectivity and shadowing, but did not assess photoreceptor metrics. Lombardo et al.^10^^,^^11^ found slightly reduced cone density at the parafovea in type 1 (T1) DM using a commercially available AOSLO system, which enables confocal imaging of the retina, and were able to differentiate between DR and absence of DR using a combination of cone metrics.^10^^,^^11^ Also using a confocal AOSLO, Lammer et al.^12^ found that regularity of the cone arrangement in both T1 and T2 DM was associated with the presence of DR, increasing DR severity and edema.

Impaired color vision has been implicated in patients with diabetes with DR, the severity of which increases with disease progression.^13^^–^^16^^,^^18^ Although the data are somewhat limited, there is evidence for changes in color vision in those with subclinical DR, even when using relatively crude screening tools, such as the Farnsworth-Munsell 100‐hue test.^18^^,^^19^ However, the predictive accuracy of conventional color vision testing does not provide support for its use as a screening tool for DR.^20^ One limitation is that such tests exhibit large variability, do not account for color threshold changes caused by normal, healthy aging, often fail to isolate the use of only red/green (RG) and yellow/blue (YB) chromatic mechanisms, and are also insensitive to small changes in chromatic discrimination.^21^

The Colour Assessment and Diagnosis (CAD test) uses random dynamic luminance modulation to isolate chromatic signals, enabling sensitive and accurate assessment of both type and severity of color vision loss.^22^^,^^23^ The CAD test has been used to determine the normal range of chromatic discrimination thresholds,^22^ as well as to demonstrate the loss of color vision that occurs during aging,^24^ enabling the establishment of normal age-adjusted limits. Using this technique, it was demonstrated that approximately 70% of patients with diabetes with no more than moderate maculopathy had abnormally high RG and YB chromatic discrimination thresholds,^25^^–^^27^ highlighting its potential as a tool for identifying patients who may be at risk of developing DR.

To date, no studies have assessed the photoreceptor layer in DR and investigated its structural parameters in relation to highly sensitive measures of visual function. Here, we use AOSLO and the CAD test to assess the relationship between cone metrics and functional measures of vision in patients with DM across all stages of retinopathy.

## Methods

This study adhered to the tenets of the Declaration of Helsinki and was approved by the local NHS review board (IRAS: 289911). Written informed consent was obtained from all patients after the nature and possible consequences of the study were explained and before any study procedures began.

### Subjects

Twenty-six patients aged 18 to 70 years with a diagnosis of T1 or T2 DM were recruited via their regularly scheduled retinal clinic appointments, online adverts, and word of mouth, along with 25 normally sighted controls without DM (Supplementary Table S1). All control participants had normal or corrected to normal visual acuity (–0.13 ± 0.12 logMAR). Participants completed a screening questionnaire, and individuals (both patients with DM and healthy controls) with bilateral macular pathology, previous retinal surgery (unrelated to DR), macula laser treatment, ocular media opacity, congenital color vision deficiency, any other systemic or ocular conditions, or an inability to consent, were excluded from participation. Individuals with such features in just one eye remained eligible: in such cases, the unaffected eye was used as the study eye. One patient had previously undergone anti-VEGF treatment for diabetic macular edema in the non-study eye. Some patients with advanced DR had undergone peripheral laser treatment but no central treatment in the study eye.

DR was graded as R0 (no retinopathy), R1 (background retinopathy), R2 (pre-proliferative retinopathy), or R3 (proliferative retinopathy), by an experienced medical retina grader (author K.A.), using wide-field color fundus photographs (Optomap, Optos).

Axial eye length was measured in each eye using the Zeiss IOL Master 700 (Carl Zeiss, Meditec) and used to scale AOSLO images. One eye was used for assessment, chosen based on best corrected visual acuity, eye dominance, or the eye without macula edema. On completion of all functional assessments, the study eye was dilated using one drop of phenylephrine hydrochloride (2.5%) and one drop of tropicamide (1%). All images were acquired post-dilation.

### Functional Assessments

Screening for congenital color vision deficiencies was achieved using the Hardy-Rand-Rittler (HRR) and the Rayleigh match (HMC Anomaloscope, OCULUS) when possible. Participants who indicated a congenital color vision deficiency in their screening questionnaire or whose HRR and/or Rayleigh match results indicated selective RG loss were excluded from the study. The HRR test uses large pseudo-isochromatic test plates illuminated with a standard daylight illuminant (D65, CIE 1931) to detect RG and YB color deficiency. The Rayleigh match uses a stimulus comprising of a circle divided into two monochromatic halves: one half is a yellow test light and the participant's task is to achieve a perceptual match with the other half, which ranges from green to red. A normal trichromat will only be able to match a narrow range of yellow test light values, whereas an individual with RG dichromacy will match the full range. The matching range and values indicate the severity and type of RG deficiency (i.e. protan or deutan), respectively.^28^^,^^29^

The CAD test was run monocularly using the Advanced Vision and Optometric Tests (AVOT) system (City Occupational Ltd.). Equipment setup, display calibration, and participant testing was carried out as previously described.^30^^,^^31^

### Colour Assessment and Diagnosis test

RG and YB chromatic sensitivity thresholds were determined using the CAD test, which was designed to isolate the use of color signals. The test uses a fully calibrated, 10-bit visual display, and the patient is adapted to a uniform background field of luminance 24 cd/m^2^ and CIE (x, y) chromaticity 0.305, 0.323 (which approximates daylight at 6500 K). The neutral background stimulus consists of a central square, comprised of 15 × 15 checks that vary randomly in luminance to generate rapid, dynamic luminance contrast noise. The colored foreground target comprises of 5 × 5 checks and subtends approximately 1 degree × 1 degree at the eye at a viewing distance of 1.4 m. During each presentation, the colored stimulus moves diagonally at approximately 3 degrees/second across the central background square through an angle of approximately 2.26 degrees. The subject's task is to indicate the end point of travel of the moving, color-defined target by pressing one of four response buttons, corresponding to the four corners of the neutral background square. The patient was instructed to look at the center of the flickering field, although the large stimulus size and displacement on the retina minimizes the need for steady fixation.

The standard CAD test uses 12 chromatic displacement directions selected to measure RG thresholds and 4 directions to measure YB thresholds. The 12 displacement directions are needed to counter intersubject variation in color confusion axes and to identify accurately deutan and protan deficiencies. The color signal strength along each direction was controlled by a staircase procedure.^23^^,^^24^

All thresholds are expressed in “Standard Normal” CAD units, calculated using data from 330 young, healthy subjects.^32^ One CAD unit (for each RG or YB) represents the median color signal thresholds for normal, young trichromats. A patient with a threshold of 3 CAD units requires 3 times greater color signal strength at threshold when compared to the standard normal CAD observer.^22^

### Structural Assessments

#### Optical Coherence Tomography Angiography

Optical coherence tomography angiography (OCTA) images of the superficial retinal vascular plexus were acquired for AOSLO alignment purposes only. OCTA images were acquired using 3 × 3 mm and 12 × 12 mm scans using the Zeiss Cirrus 5000, after dilation and before AOSLO imaging.

#### Adaptive Optics Scanning Light Ophthalmoscopy

Fixation was maintained during AOSLO imaging by instructing participants to fixate on an internal target, the position of which was controlled by the operator. Using confocal and non-confocal imaging modalities, videos were acquired centrally and along the temporal meridian using an AOSLO system housed at Moorfields Eye Hospital NHS Trust, London: either the legacy system^33^ or the quadrant-detection system.^34^ Where possible, the temporal, inferior, and superior meridians were sampled using 1.0 degree and 1.5 degree fields of view (FOVs).

Image sequences consisting of 150 frames and recorded at 16.6 frames per second were acquired at each retinal location. At least 40 of these frames were registered and averaged to produce images with a high signal-to-noise ratio,^35^ which were montaged using automated software,^36^ which outputs to Adobe Photoshop (version 13; Adobe Inc.). The adaptive optics (AO) montage was superimposed onto the OCTA image and the anatomic fovea was marked as previously described, using the superior and inferior strips to aid alignment.^37^ Each montage was produced by one person (author M.V.) and inspected for alignment by a second person (author T.K.) to ensure that no erroneous montages were created and that all montages were appropriately aligned to the foveal center using the OCTA image. Using the anatomic foveal center as an anchor, three 55 × 55 µm regions of interests (ROI) at 0.5 degrees (0.5T), 1.0 degrees (1T), and 2 degrees (2T) along the temporal meridian at each location were extracted and used to assess cone density and intercell regularity. The cones were marked in both confocal and non-confocal modalities wherever possible using a semiautomatic algorithm that marked the location of individual cones.^38^ The coordinates in each ROI were inspected twice by two masked graders (authors M.V. and N.T.), who added/removed cone markings as necessary. These coordinates were then used to calculate cone density and intercell regularity given by the bound Voronoi cells in the ROI. Statistical analysis was completed using IBM SPSS statistics (version 28; IBM, Armonk, NY, NY) and a *P* value less than 0.05 was considered statistically significant. Normality testing was used to guide the use of parametric or nonparametric statistical analysis.

## Results

Twenty (out of 26) patients with DM and 19 (out of 25) healthy controls had AOSLO confocal images that were of sufficient quality to be analyzed. Failure to acquire analyzable AOSLO images was mainly owing to the low signal-to-noise ratio, most often due to lack of patient cooperation or deterioration of tear film. The non-confocal modality could not be resolved in the majority of participants due to insufficient image quality or the mosaics being too densely packed,^33^ so cone density was assessed using the confocal modality only. Of the included AOSLO datasets, the agreement in cone density measurements between the two graders was assessed using the intraclass correlation coefficient (ICC = 0.988 [0.980–0.993]), demonstrating excellent agreement.

RG and YB CAD thresholds are not affected significantly by small residual refractive errors, aberrations, or scattered light because of the large stimulus size employed and the absence of fine spatial judgments. However, severe cataracts can lead to a significant reduction in short-wavelength light reaching the retina, and hence decreased S-cone excitation, which has been shown to cause a significant increase in YB thresholds.^24^ Two participants (both patients with DM) indicated the presence of mild cataracts in their screening questionnaires (both were imaged successfully) and yielded CAD thresholds of RG: 7.64; 6.35 and YB: 20.04; 7.73. In the absence of reliable data on the expected reduction in retinal illuminance caused by cataracts, the patients were removed from CAD-related analyses.

**Figure 1.**
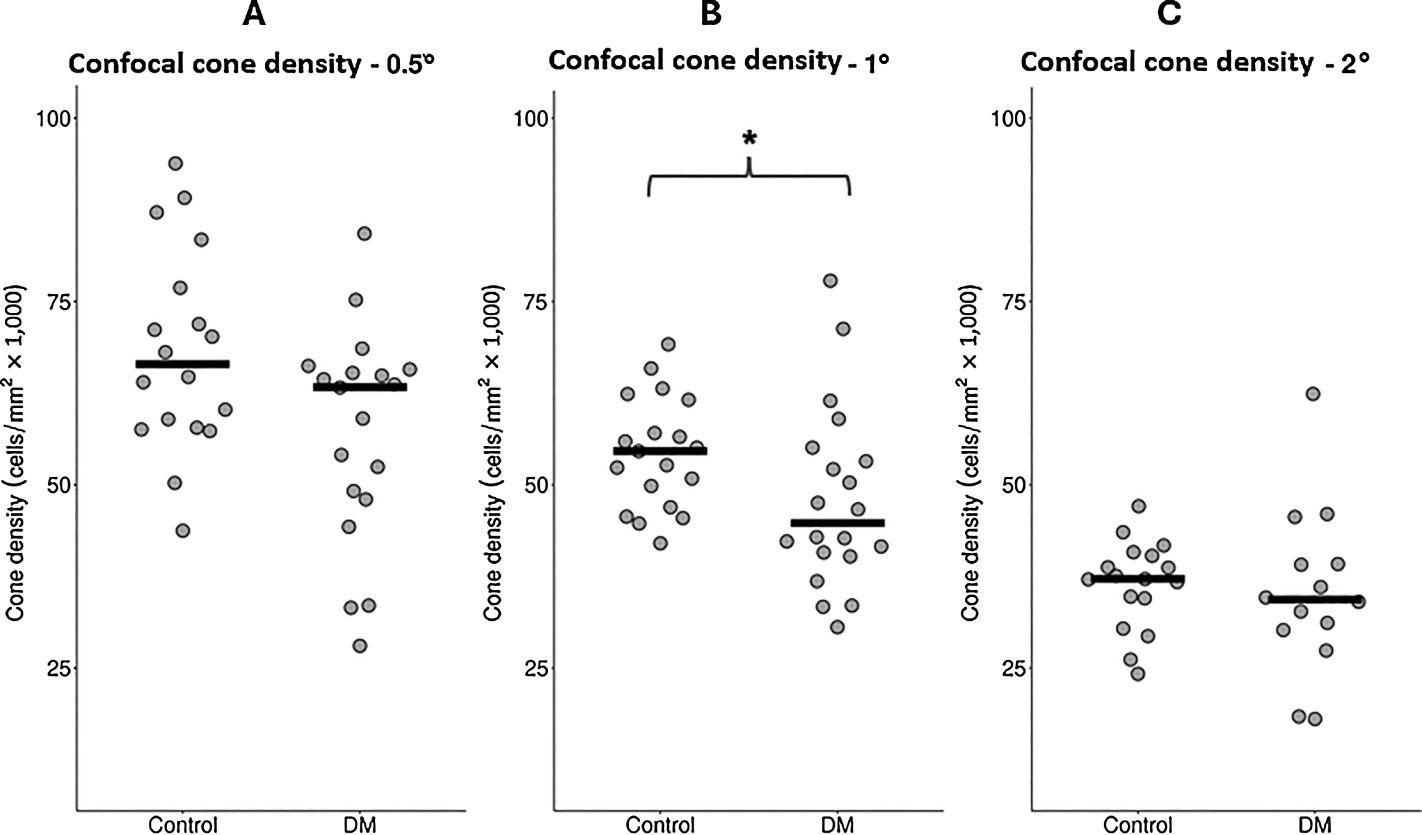
Confocal cone density at (**A**) 0.5 degrees, (**B**) 1 degree, and (**C**) 2 degrees of temporal eccentricity in patients with diabetes mellitus (DM) and healthy controls. *Horizontal lines* indicate the median; and an *asterisk* indicates a statistically significant difference.

**Figure 2.**
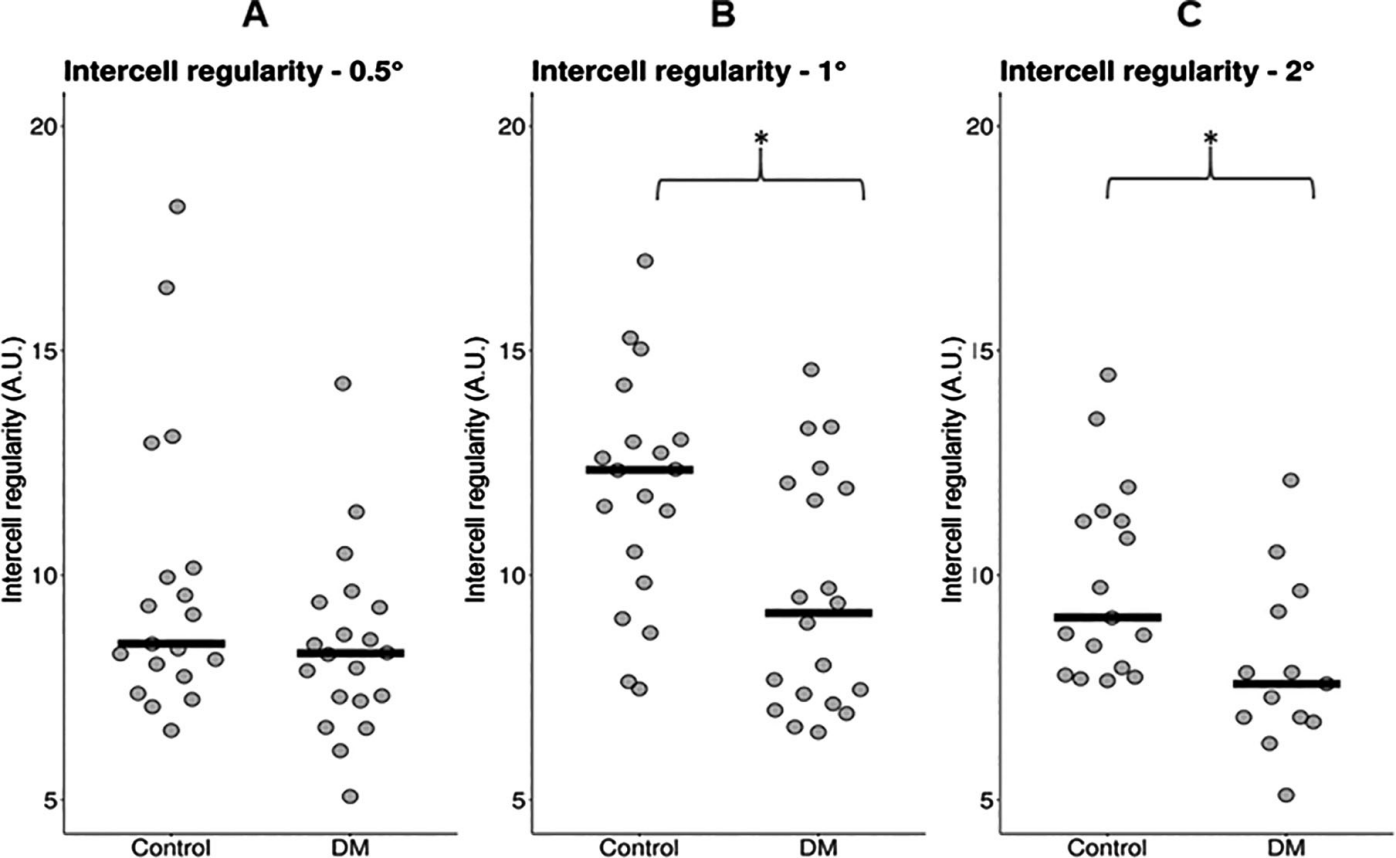
Intercell regularity in arbitrary units (A.U.) at (**A**) 0.5 degrees, (**B**) 1 degree, and (**C**) 2 degrees temporal eccentricity in patients with diabetes mellitus (DM) and healthy controls. *Horizontal lines* indicate the median; and an *asterisk* indicates a statistically significant difference.

The mean values across all cone counts for each retinal location were used for the following analysis (see the Table). Mann-Whitney *U* testing revealed significantly greater RG and YB CAD thresholds, as well as lower cone density at 1T (but not 0.5T or 2T, Fig. 1) and reduced intercell regularity at 1T and 2T (but not 0.5T, Fig. 2) in patients with DM than in healthy controls. When stratified according to the DR grade, Kruskal-Wallis testing revealed no significant differences between the groups in color vision thresholds (RG: *P* = 0.900 and YB: *P* = 0.314), cone density values (0.5T: *P* = 0.429; 1T: *P* = 0.347; and 2T: *P* = 0.134) or intercell regularity (0.5T: *P* = 0.371; 1T: *P* = 0.249; and 2T: *P* = 0.277).

**Table.** Colour Assessment and Diagnosis (CAD) Thresholds and Confocal Cone Metrics Analysis in Healthy Controls and Patients With DM

		Controls			Patients With DM			
		Median	IQR	*n*	Median	IQR	*n*	*P* Value
**CAD thresholds**	**RG**	1.24	0.37	24	2.77	1.69	20	<0.001^*^
	**YB**	1.51	0.47	25	3.45	2.33	20	<0.001^*^
**Confocal cone density, cells/mm^2^**	**0.5T**	66,390	17,554	18	63,462	17,000	20	0.126
	**1T**	54,574	10,938	19	44,744	13,044	20	0.024^*^
	**2T**	37,143	5,800	17	34,324	8,742	14	0.493
**Intercell regularity**	**0.5T**	8.47	2.17	19	8.26	2.04	20	0.235
	**1T**	12.34	2.82	19	9.16	4.67	20	0.013^*^
	**2T**	9.05	3.27	17	7.58	2.35	14	0.012^*^

* Statistically significant

All cone metrics are corrected using the area bound by cones to eliminate edge-artifacts. The two patients with DM with cataracts were removed from the CAD analysis to avoid the potential confounding effects of lens yellowing.

DM, diabetes mellitus; IQR, interquartile range; RG, red-green; YB, yellow-blue.

An independent *t*-test revealed a significant difference in age between the DM and control groups (see the Table). The analysis was therefore repeated, while controlling for age, using 1-way ANCOVA. However, it is worth noting that with increasing age there is only a small increase in CAD thresholds (approximately 1% increase per year for RG and approximately 1.6% increase/year in YB thresholds)^39^ and only a small decrease in cone density (approximately 116 cones/mm^2^ per year).^39^ The difference between groups remained statistically significant for both RG (*F* (1, 41) = 21.937, *P* = 0.002, partial η^2^ = 0.214) and YB (*F* (2, 42) = 38.351, *P* < 0.001, partial η^2^ = 0.278) CAD thresholds. In contrast, there were no significant differences between patients with DM and healthy controls in cone density at any of the investigated locations (0.5T: *P* = 0.716; 1T: *P* = 0.385; and 2T: *P* = 0.393). There remained, however, a statistically significant difference between groups in intercell regularity at 1T (*F* (1, 36) = 5.753, *P* = 0.022, partial η^2^ = 0.138), but not at 0.5T (*P* = 0.573) or 2T (*P* = 0.11).

**Figure 3.**
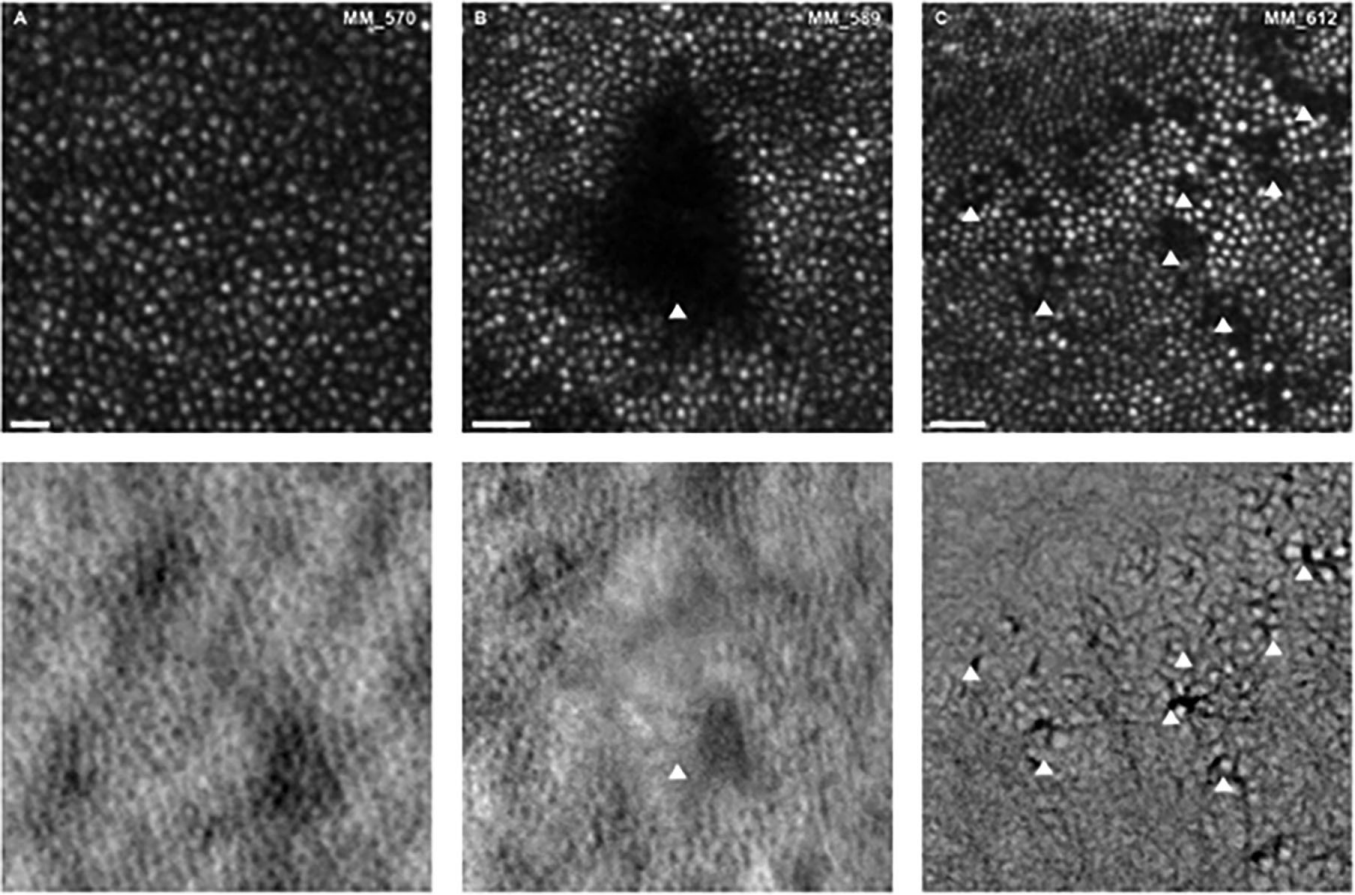
AOSLO images of the cones in (**A**) a healthy control, for comparison with examples of abnormal reflectivity (*arrows*, **A** and **B**) observed in confocal (*top*) and non-confocal images from patients with diabetes mellitus. Split-detection was used to image **A** and **B** and quadrant-detection was used to image **C** (*bottom row*). Location of **A** is approximately 1.5 degrees superior, **B** is approximately 1.5 degrees inferior, and **C** is approximately 0.5 degrees nasal from the fovea. Scale bars are 20 microns.

In addition to the quantitative findings, localized areas of abnormal reflectivity were observed in some patients with DM (Fig. 3).

**Figure 4.**
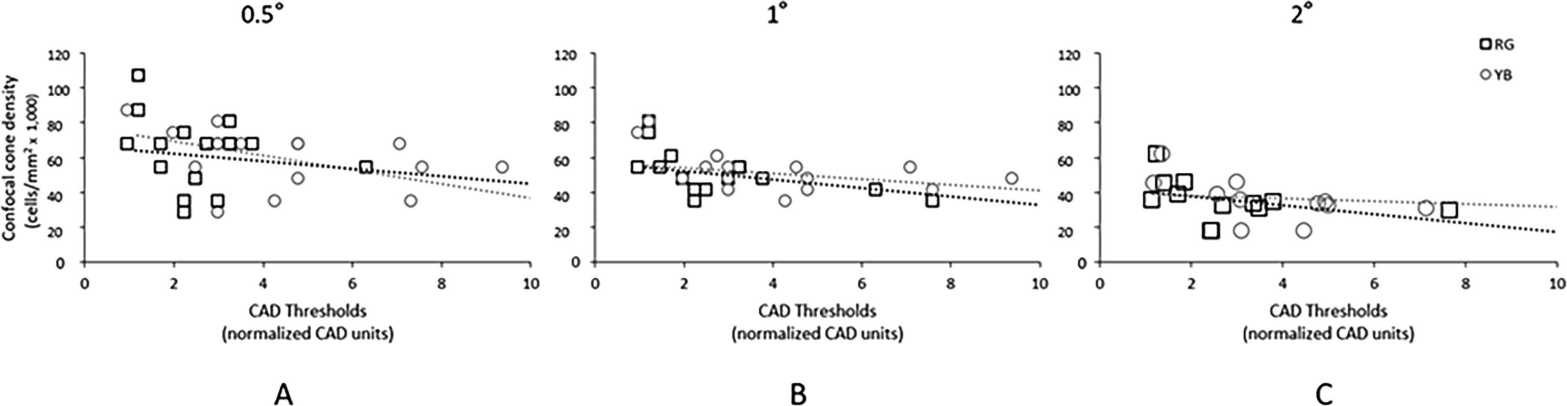
Relationship between both red-green (RG, *black squares*) and yellow-blue (YB, *gray circles*) Colour Assessment and Diagnosis (CAD) test thresholds and confocal cone density at (**A**) 0.5 degrees, (**B**) 1 degree, and (**C**) 2 degrees temporal eccentricity in patients with diabetes mellitus. The rightmost RG and YB data points in **A** and **B** represent the same participant (MM_612, see Supplementary Table S1, Fig. 3C), whose screening results were indicative of general, as opposed to congenital, color vision loss (this patient did not have analyzable data at 2 degrees). The two patients with DM with cataracts were removed from the CAD analysis to avoid the potential confounding effects of lens yellowing.

A Spearman correlation revealed statistically significant inverse correlations between cone density at 0.5T (*r_s_* (16) = −0.609, *P* = 0.012), 1T (*r_s_* (14) = −0.552, *P* = 0.041), and 2T (*r_s_* (11) = −0.718, *P* = 0.013) and YB CAD thresholds in patients with DM. However, there was no statistically significant correlation between cone density at any location and RG CAD thresholds in patients with DM (Fig. 4). No relationship between cone density and CAD thresholds was evident in the control group at 0.5T (RG: *r_s_* (17) = 0.064, *P* = 0.808; YB: *r_s_* (18) = 0.100, *P* = 0.693), 1T (RG: *r_s_* (18) = 0.040, *P* = 0.874; and YB: *r_s_* (19) = 0.332, *P* = 0.166), or 2T (RG: *r_s_* (16) = −0.424, *P* = 0.102 and YB: *r_s_* (17) = −0.255, *P* = 0.323). There was no relationship between intercell regularity and RG or YB thresholds in either group (all *P* > 0.142).

## Discussion

### Color Vision in Patients With DM Versus Healthy Controls

In line with previous work, we found that patients with DM had significantly higher color vision thresholds compared to visually healthy control subjects.^25^^,^^26^ This finding persisted even after controlling for age differences between groups. In fact, 16 of our 23 patients with DM (but no cataracts) had RG and/or YB CAD thresholds outside of the normal limits^24^; 3 of whom were patients who had no DR (R0) according to their fundus photography. Chromatic signals are pooled and summed over relatively large areas of the retina, particularly in the CAD test which requires the subject to judge changes in the stimulus position in order to extract the correct direction of motion.^24^ If any of the retina within a diagonal distance of ±2 degrees ends up with reduced cone density, the corresponding color threshold is likely to increase as a result of being more difficult to judge the direction of motion. Therefore, this may account for the higher CAD thresholds seen in our patients with DM. These findings support the hypothesis that functional changes can precede visualization of structural changes in DR using commercially available imaging tools (such as fundus photography and optical coherence tomography [OCT]), indicating that significant cell loss may occur before visual abnormalities are noticed by the patient.^40^

### Confocal Cone Density and Regularity in Patients With DM Versus Healthy Controls

Confocal cone density was found to be lower at 1T in patients with DM than in healthy controls, although the difference was not significant at 0.5T nor 2T. Intercell regularity was found to be lower at 1T and 2T in patients with DM than in healthy controls, but not at 0.5T. After controlling for age, only regularity at 1T remained significantly different between groups.

The lack of a statistical difference in cone density at either 0.5T or 2T is most likely owing to naturally high variability,^41^ tight cone packing (and resultant challenges in visualizing and quantifying cells in the foveal region), insufficiencies in the analysis method used (discussed later), or potentially low sample size.

Unfortunately, with the current data, it is not possible to determine whether the lower cone density we found in our patients with DM is representative of cone absence or simply lack of waveguiding outer segments,^42^ as not all cones could be resolved using non-confocal imaging. However, the accompanying changes in intercell regularity suggest some extent of cone loss. Ongoing improvements in imaging, such as quadrant-detection, and analysis techniques may help to improve the visibility of cones in non-confocal images (particularly at the fovea, where they are most densely packed), which may enable us to examine the structural integrity of cone inner segments in these patients and the relationship with color vision.

### Structure-Function Relationship

The CAD test and AOSLO are currently being used as outcome measures in phase I/II clinical trials (e.g. NCT02599922 and NCT01846052) for gene therapy, but have not yet been validated against each other. This is the first study to investigate the relationship between these two outcome measures in people with DM. Crucially, we found a significant structure-function relationship – with lower confocal cone density being associated with poorer YB color sensitivity – for patients with DM. This relationship was not observed in healthy controls, indicating that this may be a feature unique to patients with acquired retinal damage, and could have important implications for how cone integrity affects vision in progressive eye disease. The relationship was not observed in intercell regularity for either people with DM or healthy controls, suggesting that a regular arrangement of cones may not be critical to retention of visual function. However, longitudinal imaging and vision testing would be needed to ascertain whether there is any effect of cone rearrangement (following loss) on visual function. Future work investigating the structure-function relationship in other diseases is also warranted to ascertain if the density-color relationship is applicable to conditions other than DM.

The finding that both RG and YB color vision is affected in DM may suggest that there is generalized cone loss in DR. As we did not have the means to assess photopigment densitometry, we cannot postulate which cone types are non-waveguiding in our images. The question as to whether S-cones are damaged earlier than L- and M-cones in DM is of interest, because it is well-established that loss of YB color vision in DR precedes RG.^43^ A key finding from the current data is that the dysflective/absent cones most often appear to be adjacent to each other, which is not consistent with S-cone topography.^44^ As such, it is unlikely that abnormalities are isolated to S-cones, although it is possible that primary death of S-cones causes secondary death of neighboring cones in a mechanism known as the “bystander effect.”^45^ Alternatively, the earlier/greater loss in YB color vision could simply result from the naturally lower ratio of S-cones to L/M-cones, as an equivalent loss of cones would represent a higher proportion in this subtype.

### Areas of Dysflective Cones

In line with previous work,^46^ areas of dysflective cones were observed in patients with DR using confocal imaging. Without the corresponding non-confocal images it is not, however, possible to ascertain whether these areas are indicative of cone loss, altered waveguiding, or simply normal variation in cone reflectivity.^6^^,^^47^^,^^48^ It is possible that the dysflective cones are a result of hypoxia – a common occurrence in retinas of patients with DR.^49^ An additional explanation is that the dark areas are caused by shadows cast onto the photoreceptor layer by, for example, floaters (vitreous humor), edema, cysts, or debris (inner retina). Although this is a plausible explanation for the pattern of reduced reflectivity in Figure 3B, the punctate hyporeflectivity (dark spots) observed in Figure 3C is highly localized, suggesting photoreceptor origin. One complication is that changes in reflectivity occur in the normal retina due to temporal fluctuation and in response to light stimulation,^50^^,^^51^^,^^5^ highlighting the need for longitudinal imaging in patients with cone abnormalities.^36^

Quantification of dysflective cones is difficult to achieve using current methods, as their locations vary and may not fall within the ROIs that were predefined as part of our protocol (0.5T, 1T, and 2T, in this instance). This may explain why we observed only moderate differences in cone density/regularity between those with DM and those without. It is therefore of interest in future work to adopt alternative analysis techniques, such as artificial intelligence – particularly those that assess the mosaic across the entire montage. Such analysis techniques would likely make the analysis more efficient and could potentially provide more information on the mechanism and pattern of cone loss in people with DM. In addition, assessment of cone inner segment integrity (using non-confocal imaging), as well as alignment with and analysis of other imaging modalities (such as OCT, OCTA, or fundus images) may help to elucidate the origin of the dark areas observed in confocal AOSLO images. Future work investigating retinal thickness and the foveal avascular zone, as well as their relationship to color vision thresholds, is warranted.

## Conclusions

DR has long been regarded as a vascular disease; however, in line with recent evidence, we have shown that the photoreceptors are also altered in DR, which has a direct impact on visual function. It is well known that photoreceptor death is irreversible, highlighting the need for sensitive screening techniques both to facilitate early detection and to monitor progression of disease and/or efficacy of treatment. Larger sample sizes with greater representation across DM disease stage is needed for more definitive conclusions; however, the significant relationship found between CAD and cone metrics in this study shows promise for early detection of changes, potentially before patients become aware of visual disturbances or present in the clinic. Such findings highlight that the photoreceptors may serve as a means for screening and monitoring systemic disease, having critical implications for future oculomics research.

In conclusion, these findings suggest that, in patients with DM, loss of cone reflectivity, and likely structure, is accompanied by a proportional decrease in YB chromatic discrimination sensitivity. Further research is warranted to determine whether this structure-function relationship also extends to other retinal and systemic diseases.

## Supplementary Material

Supplement 1
