# Supplementary material for: Changes in Waveguiding Cone Photoreceptors and Color Vision in Patients With Diabetes Mellitus
Source: Invest Ophthalmol Vis Sci. 2024 Dec 13;65(14):28. doi: 10.1167/iovs.65.14.28 (PMC11645757; doi:10.1167/iovs.65.14.28)
Supplement: Supplement 1 [file iovs-65-14-28_s001.pdf]

| ID | Condition | Study eye | Age | Gender | Notes |
|---|---|---|---|---|---|
| MM_0597 | Control | OD | 24 | Female | |
| MM_0566 | Control | OS | 26 | Female | |
| MM_0569 | Control | OD | 27 | Female | Unsuccessful AOSLO |
| MM_0564 | Control | OS | 30 | Female | |
| MM_0563 | Control | OD | 31 | Female | |
| MM_0639 | Control | OS | 33 | Female | |
| MM_0578 | Control | OS | 34 | Female | |
| MM_0626 | Control | OD | 34 | Female | |
| MM_0570 | Control | OS | 36 | Female | |
| MM_0584 | Control | OS | 38 | Female | Unsuccessful AOSLO |
| MM_0645 | Control | OD | 40 | Female | |
| MM_0592 | Control | OD | 43 | Female | Unsuccessful AOSLO |
| MM_0583 | Control | OD | 59 | Female | Unsuccessful AOSLO |
| MM_0624 | Control | OS | 59 | Female | |
| MM_0562 | Control | OD | 24 | Male | Unsuccessful AOSLO |
| MM_0363 | Control | OS | 27 | Male | |
| MM_0574 | Control | OD | 27 | Male | |
| MM_0364 | Control | OD | 28 | Male | |
| MM_0596 | Control | OS | 29 | Male | |
| MM_0603 | Control | OD | 29 | Male | |
| MM_0573 | Control | OS | 30 | Male | |
| MM_0568 | Control | OD | 31 | Male | |
| MM_0254 | Control | OD | 40 | Male | |
| MM_0654 | Control | OS | 67 | Male | |
| MM_0575 | Control | OD | 69 | Male | Unsuccessful AOSLO |
| MM_0589 | DM | OD | 24 | Female | |
| MM_0656 | DM | OD | 32 | Female | |
| MM_0605 | DM | OS | 48 | Female | |
| MM_0606 | DM | OS | 62 | Female | |
| MM_0653 | DM | OS | 65 | Female | Unsuccessful AOSLO |
| MM_0601 | DM | OD | 66 | Female | Mild cataracts |
| MM_0612 | DM | OD | 37 | Male | |
| MM_0609 | DM | OD | 41 | Male | Unsuccessful AOSLO |
| MM_0640 | DM | OD | 44 | Male | |
| MM_0621 | DM | OS | 46 | Male | Mild cataracts; peripheral laser |
| MM_0615 | DM | OD | 47 | Male | |
| MM_0620 | DM | OD | 48 | Male | Unsuccessful AOSLO |
| MM_0632 | DM | OD | 49 | Male | |
| MM_0595 | DM | OD | 56 | Male | |
| MM_0602 | DM | OS | 58 | Male | |
| MM_0646 | DM | OD | 58 | Male | |
| MM_0655 | DM | OD | 58 | Male | |
| MM_0616 | DM | OD | 60 | Male | Peripheral laser; anti-VEGF in non-study eye |
| MM_0649 | DM | OD | 60 | Male | |

| | | | | | |
|---|---|---|---|---|---|
| MM_0618 | DM | OD | 61 | Male | |
| MM_0650 | DM | OD | 62 | Male | |
| MM_0599 | DM | OD | 63 | Male | |
| MM_0635 | DM | OD | 65 | Male | Unsuccessful AOSLO |
| MM_0636 | DM | OD | 65 | Male | Unsuccessful AOSLO |
| MM_0611 | DM | OD | 67 | Male | Unsuccessful AOSLO |
| MM_0637 | DM | OD | 69 | Male | |

Supplementary Table 1. Demographics of included participants.

DM = patients with diabetes mellitus; OD = right eye; OS = left eye.
